# Trends in PCSK9 Inhibitor Prescriptions before and after the Price Reduction in Patients with Atherosclerotic Cardiovascular Disease

**DOI:** 10.3390/jcm10173828

**Published:** 2021-08-26

**Authors:** Alex Smith, Drew Johnson, Joshua Banks, Scott W. Keith, Dean G. Karalis

**Affiliations:** 1 Washington Hospital Center, MedStar Georgetown University, Washington, DC 20010, USA; alexander.smith0159@gmail.com; 2 Department of Cardiology, Sidney Kimmel Medical College, Thomas Jefferson University Hospital, Philadelphia, PA 19107, USA; drew.johnson@jefferson.edu; 3 Department of Pharmacology and Experimental Therapeutics, Division of Biostatistics, Sidney Kimmel Medical College, Thomas Jefferson University, Philadelphia, PA 19107, USA; Joshua.Banks@jefferson.edu (J.B.); Scott.Keith@jefferson.edu (S.W.K.)

**Keywords:** lipids, atherosclerotic cardiovascular disease, PCSK9 inhibitors

## Abstract

Background: Proprotein convertase subtilisin kexin type 9 (PCSK9) inhibitors reduce low-density lipoprotein (LDL) cholesterol and cardiovascular event rates, yet due to their high price remain underutilized and difficult to prescribe in clinical practice. In March 2018, their price was significantly reduced. We evaluated whether the price reduction would improve prescribing patterns of PCSK9 inhibitors in eligible patients with atherosclerotic cardiovascular disease (ASCVD). Methods: We identified the number of eligible ASCVD patients and those prescribed a PCSK9 inhibitor for each year between July 2015 and December 2019. Patient demographics and clinical characteristics for those prescribed a PCSK9 inhibitor were extracted from their electronic health record. Results: In total 1059 patients of eligible patients received a new prescription for a PCSK9 inhibitor. From 2015 to 2019, the rate of new prescriptions among eligible patients increased from 0.5 to 3.3% (*p* < 0.001) and continuation rates increased from 18 to 60% (*p* < 0.001). Following the price reduction, patients who were prescribed a PCSK9 inhibitor were younger and more likely to be female, but less likely to have Medicare insurance. Conclusions: Despite the reduction in the cost of PCSK9 inhibitors, most eligible patients are not prescribed one. The reduction in cost has improved adherence, primarily in patients with commercial insurance. Older patients and those on Medicare still face significant barriers in accessing a PCSK9 inhibitor. Further reductions in the price of the PCSK9 inhibitors are needed as is further study of the barriers that exist in prescribing one.

## 1. Introduction

In 2015, the United States (US) Food and Drug Administration approved the proprotein convertase subtilisin kexin type 9 (PCSK9) inhibitors alirocumab and evolocumab for the treatment of hypercholesterolemia in patients with atherosclerotic cardiovascular disease (ASCVD) or familial hypercholesterolemia, who need additional low-density lipoprotein (LDL) cholesterol lowering. When added to a maximally tolerated statin alone or in combination with ezetimibe, PCSK9 inhibitors significantly lower LDL cholesterol by up to 60% and do so safely and with few adverse effects [1]. In large, randomized outcome studies in patients with clinical ASCVD, both evolocumab [2] and alirocumab [3] have demonstrated improved cardiovascular outcomes. Despite their safety and cardiovascular benefits, they have been underutilized in clinical practice. In one study among 368,624 PCSK9 inhibitor eligible patients almost 2 years after PCSK9 inhibitors became available, only 1753 (<0.05%) received a PCSK9 inhibitor prescription [4]. The initial cost of these medications was high, with an average cost of over 14,000 USD (US dollars) per year. This led to difficulty in obtaining payor approval. Denials were frequent due to stringent eligibility criteria and difficult and time-consuming pre-authorization processes [5]. Up to 63% of prescriptions were rejected by insurance companies and many prescriptions that were approved were not collected or filled due to the high out-of-pocket patient costs and co-pays [6].

At the 2015 prices, the PCSK9 inhibitors were determined not to be cost efficient and it was estimated that the cost would need to be reduced to an average of 4500 USD per year to meet the threshold of cost-effectiveness [7]. In March of 2018, the cost of alirocumab was substantially reduced to an average cost of up to 5850 USD per year, and soon after the cost of evolocumab was similarly reduced. Even at the discounted price, subsequent cost-effective analyses found the PCSK9 inhibitors in general not to be cost-effective [8,9,10]. The aim of this study was to evaluate prescribing patterns of PCSK9 inhibitors, and the characteristics of patients prescribed one before and after the price reduction for real-world patients.

## 2. Methods

The study population was selected from a large single-specialty cardiology practice, Cardiology Consultants of Philadelphia (CCP). CCP is the largest independent cardiac care practice in the US and has 32 cardiology centers and 97 cardiologists across the Delaware Valley and Philadelphia regions. All the cardiologists practice adult cardiology and manage general cardiology patients, and hence are expected to follow guideline-based therapy for the secondary prevention of ASCVD. The centers provide care in both urban and suburban locations and have cardiologists based in both academic and community settings. Patients were included in the study if they had a diagnosis of coronary artery disease, cerebrovascular disease, or peripheral arterial disease in their electronic health record (EHR) problem list, as defined by the 9th and 10th International Classification of Diseases (ICD) codes. EHR data were analyzed from July 2015 (the time when the first PCSK9 inhibitor became clinically available) to December 2019. Data were queried from the practice EHR database, not by individual chart review.

The patient cohort comprised the 1059 patients who were prescribed a PCSK9 inhibitor between July 2015 and December 2019. The study was approved by the independent review board at Thomas Jefferson University Hospital. Patient demographics and clinical characteristics were extracted from the EHR data. A diagnosis of hypertension or diabetes was ascertained from the ICD codes in the problem list. In addition, a patient was considered to have diabetes if they were on a medication used to treat diabetes. Cigarette smoking was extracted from the EHR and was self-reported. For patients prescribed a PCSK9 inhibitor, the LDL-cholesterol level at baseline before being prescribed a PCSK9 inhibitor was recorded. Lipid lowering medications were recorded from the patient’s EHR medication list. When a PCSK9 inhibitor was added for the first time in a patient’s medication list it was considered a new prescription. Patients were considered to have continued their PCSK9 inhibitor prescription if it remained on their active medication list and have discontinued it if it had been removed from their active medication list. The statin dose was recorded from the patient’s last visit. High-intensity statin was defined as 40 or 80 mg of atorvastatin or 20 or 40 mg of rosuvastatin. For each year, from 2015 to 2019, we identified the number of patients who were eligible for a PCSK9 inhibitor based on a diagnosis code for ASCVD, an LDL-cholesterol ≥ 70 mg/dL, and the patient having at least one office visit within the year of interest.

## 3. Statistical Analysis

Chi-squared tests were used to compare patient characteristics between PCSK9 inhibitor users who initiated their PCSK9 inhibitor before 1 March 2018 to those initiating after 1 March 2018, the date assigned to the price reduction for both PCSK9 inhibitors. We constructed a multivariable logistic regression model to compare those receiving their first PCSK9 inhibitor prescription after the price change versus those receiving their first PCSK9 inhibitor before the price change. The predictor variables included age, gender, insurance type, diabetes, ezetimibe use, smoking, hypertension, statin use, and LDL cholesterol level. The numbers of PCSK9 inhibitor eligible patients, numbers of physicians in the practice, and the numbers of new PCSK9 inhibitor prescriptions per year from 2015 to 2019 were collected and tested for a time trend using a Poisson regression model of the prescription counts, offset by the log of the number of patients. The nominal significance level for each test was set in advance to α = 0.05. All statistical analyses were conducted using SAS v9.4 (SAS Institute, Cary, NC, USA).

## 4. Results

**Figure 1:**
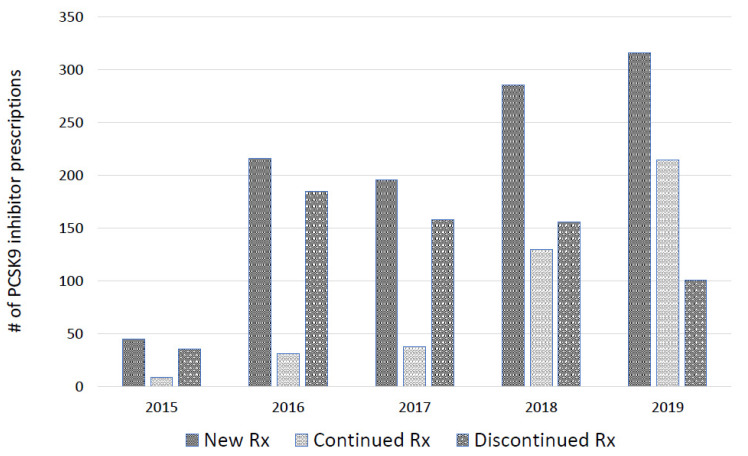
Number of new, continued, and discontinued PCSK9 inhibitor prescriptions from 2015 to 2019. # = number; Rx = prescription; PCSK9 = proprotein convertase subtilisin kexin type 9.

From 2015 to 2019, 1059 patients with ASCVD received a new prescription for a PCSK9 inhibitor. The characteristics of the patients are presented in Table 1. Among these PCSK9 inhibitor patients, 66% were over the age of 65 and 47.3% were female. Trends in the numbers of PCSK9 inhibitor eligible patients and new PCSK9 inhibitor prescriptions are shown in Table 2. There was a statistically significant trend over time suggesting that the rate of new PCSK9 inhibitor prescriptions increased over time (*p* < 0.001) from 45 (0.26%) in 2015 to 316 (1.6%) in 2019. The number of newly prescribed patients who continued or discontinued PCSK9 inhibitor therapy from 2015 to 2019 is presented in Figure 1. There was a significant increase in PCSK9 inhibitor prescriptions after the price reduction from 158 prescriptions/year to 305 prescriptions/year (*p* < 0.001). Continuation rates of PSCK9 inhibitor prescriptions significantly increased after the price reduction from 18 to 60% (*p* < 0.001).

After the price reduction for the PCSK9 inhibitors in March 2018, patients prescribed a PCSK9 inhibitor were younger, on average with an increase in the number of patients under the age of 65 (39% post-price reduction compared with 29% pre-price reduction), and more likely to be female. In addition, after the price reduction, fewer Medicare patients were prescribed a PCSK9 inhibitor (51% post-price reduction compared with 61% pre-price reduction). Multivariable analysis suggested the odds of receiving a prescription for a PCSK9 inhibitor after the price reduction increased in younger patients and those with an LDL cholesterol of between 100 and 130 mg/dL (Table 3).

## 5. Discussion

Our study provides a current analysis of PCSK9 inhibitor use among real-world patients with ASCVD. During the study period from 2015 to the end of 2019, there was only a small increase in the number of eligible patients prescribed a PSCK9 inhibitor. The trend in the increase in new prescriptions for PCSK9 inhibitors predated the price reduction. In March of 2017, one year before the price reduction in alirocumab was announced, the results of the FOURIER trial of evolocumab were presented and demonstrated a 15 to 20% relative reduction in major cardiovascular events compared with the placebo in patients with ASCVD and other cardiovascular risk factors [2]. This led to both the American College of Cardiology and the National Lipid Association publishing recommendations in 2017 for the use of PCSK9 inhibitors in ASCVD patients [11,12]. It is likely that the positive results of the FOURIER trial and the subsequent positive results of the ODYSSEY OUTCOMES trial [3] with alirocumab, the publication of recommendations from national professional organizations, and the price reduction explain the increased prescribing of PCSK9 inhibitors in our cohort of patients with ASCVD. In addition, the price reduction was associated with a significant decrease in the PCSK9 inhibitor discontinuation rate. These findings suggest that once a PCSK9 inhibitor is prescribed, the reduction in cost has made it easier for patients to be approved and maintain PCSK9 inhibitor therapy. However, prescriptions for a PCSK9 inhibitor remain low. Previous studies using real-world EHR data found that early use of PCSK9 inhibitors was low, with <1% of eligible patients being prescribed a PCSK9 inhibitor [4,13]. Our study provides more current data on the use of PCSK9 inhibitors. Since the price reduction and positive outcome studies for both PCSK9 inhibitors and the guidelines for their use, outlined in the 2018 AHA/ACC Multisociety cholesterol guidelines [14], there have been better continuation rates of these medications, but only a small increase in new PCSK9 inhibitor prescriptions. In our study, only a small number of ASCVD patients eligible for a PCSK9 inhibitor were prescribed one. This may be related to barriers other than those imposed by the payors. Physician reluctance to prescribe a PCSK9 inhibitor may be due to past poor experiences, perceived difficulties in the pre-authorization process, or belief that at their current prices, they remain cost-inefficient. In addition, in contrast to high-intensity statin therapy, PCSK9 inhibitors do not lower hs-C-reactive protien [15]. Despite their ability to significantly lower LDL-cholesterol, the lack of an anti-inflammatory effect may have led some physicians to question the benefit of adding a PCSK9 inhibitor to a patient’s current statin therapy. In addition, patient-related factors such as high copays may explain the continued underutilization of PCSK9 inhibitors in real-world practice [16]. In a study of patients referred to a preventive cardiology clinic, approximately one-third of patients refused a PCSK9 inhibitor for economic reasons [17].

Following the price reduction, patients prescribed a PCSK9 inhibitor were younger, more likely to be female, and more likely to have commercial insurance. However, following the price reduction, fewer older patients and those on Medicare were prescribed a PCSK9 inhibitor. An analysis of nationwide Part D Medicare drug plans showed that most plans cover PCSK9 inhibitors [18]. Although covered, the high out-of-pocket costs that approach $5000 annually for PCSK9 inhibitors represent a significant barrier to prescribing and adherence in this patient population. There is a healthcare cost for those individuals who are denied access to or cannot afford a PCSK9 inhibitor. In an observational study of 139,036 individuals prescribed a PCSK9 inhibitor, the risk of a cardiovascular event was significantly higher in those individuals whose PCSK9 inhibitor prescription was either rejected or abandoned compared with those in whom the prescription was paid for [19]. Although manufacturing processes for therapeutic monoclonal antibodies have improved over the last several decades, their production costs remain significantly higher than other pharmacologic therapies. For this reason, it may be difficult to lower the cost of the monoclonal PCSK9 inhibitors to much lower levels. However, improvement in plant design and product development strategies may make the production of these agents more cost-effective and allow further reductions in their price [20].

## 6. Limitations

This study has several limitations. Medication prescriptions were identified from the patient’s EHR, and we could not determine medication adherence. We could not determine whether patients abandoned versus discontinued their PCSK9 inhibitor prescription or the reasons why. Among patients with ASCVD there is heterogeneity of risk. The 2018 AHA/ACC/Multisociety cholesterol guidelines recommend a PCSK9 inhibitor for very-high-risk ASCVD patients whose LDL-cholesterol is ≥70 mg/dL on maximally tolerated statin therapy, with or without ezetimibe. We could not categorize the ASCVD patients in our study as high compared with very high risk, and it is possible that physicians may have not prescribed a PCSK9 inhibitor in a patient who was eligible based on their treated LDL cholesterol level because they felt the patient was not particularly high risk. In addition, we did not collect data on Lp(a) which if elevated may have influenced a physician’s decision to prescribe a PCSK9 inhibitor. Despite these limitations, this study has several strengths. The study included a large cohort of ASCVD patients treated by a cardiologist and provide up-to-date findings regarding PCSK9 prescriptions since the price reduction. Although the study was conducted from a single practice, the large number of cardiologists and their diversity in practice location and type likely represent a wider group of cardiologists.

## 7. Conclusions

Despite the reduction in the cost of PCSK9 inhibitors, most eligible ASCVD patients are not prescribed one. The reduction in cost has improved adherence, primarily in patients with commercial insurance; however, most eligible patients including older patients and those on Medicare still face significant barriers in accessing a PCSK9 inhibitor. New LDL-cholesterol agents will compete with the currently available monoclonal PCSK9 inhibitors and potentially lead to further price reductions. Bempedoic acid, which inhibits ATP-citrate lyase, lowers LDL cholesterol by approximately 20% and is currently approved for high-risk patients both in Europe and in the US. Inclisiran, which is a small interfering RNA LDL cholesterol-lowering therapy, has been shown to be as effective in lowering LDL cholesterol and with a similar safety profile as the currently available monoclonal PCSK9 inhibitors. In contrast to the monoclonal PCSK9 inhibitors which are administered every 2 weeks or monthly, inclisiran is dosed every 6 months and if it is administered in a clinic or physician’s office as planned it will improve compliance and make it more cost-effective than the monoclonal PCSK9 inhibitors. Based on a series of phase III trials, inclisiran has been approved in Europe and is awaiting approval in the US. Irrespective of the impact of these new LDL-cholesterol lowering agents on the cost of the currently available monoclonal PCSK9 inhibitors, further reductions in the price of the currently available monoclonal PCSK9 inhibitors are needed, as is further study of the barriers that exist to improve access to the many patients who would benefit from these agents to further lower their LDL cholesterol and cardiovascular risk.

## Figures and Tables

**Table 1:** Baseline Characteristics of Patients Prescribed a PCSK9 Inhibitor.

Parameter	PCSK9i Prescribed	PCSK9i Prescribed Pre-Price Reduction	PCSK9i Prescribed Post-Price Reduction	*p*-Value
Total	1059	500	559	
Age (mean, std)	68.4 (9.7)	69.7 (10.0)	67.0 (9.4)	<0.001
Age				
<65	360 (34.0)	144 (28.8)	216 (38.6)	<0.001
65–71	303 (28.6)	134 (26.8)	169 (30.2)	
72–79	265 (25.0)	137 (27.4)	128 (22.9)	
≥80	131 (12.4)	85 (17.0)	46 (8.2)	
Gender				0.017
Male	558 (52.7)	256 (51.2)	245 (43.8)	
Female	501 (47.3)	244 (48.8)	314 (56.2)	
ASCVD				0.377
CAD	757 (71.5)	353 (70.6)	404 (72.3)	
CVD	47 (4.4)	25 (5.0)	22 (3.9)	
PAD	18 (1.7)	8 (1.6)	10 (1.8)	
Insurance				
Medicaid	81 (7.7)	39 (7.8)	42 (7.5)	0.006
Medicare	586 (55.3)	303 (60.6)	283 (50.6)	
Commercial	386 (36.5)	155 (31.0)	231 (41.3)	
Other	6 (0.6)	3 (0.6)	3 (0.5)	
LDL-C (mg/dL)	160.0 (55.5)	163.0 (53.1)	157.2 (57.6)	0.095
≥70 to 99	59 (5.8)	21 (4.3)	38 (7.2)	0.306
100 to 129	238 (23.5)	109 (22.5)	129 (24.3)	
130 to 159	249 (24.5)	121 (25.0)	128 (24.1)	
160 to 189	206 (20.3)	100 (20.7)	106 (20.0)	
≥190	263 (25.9)	133 (27.5)	130 (24.5)	
Statin Intensity				
High	264 (24.9)	125 (25.0)	139 (24.9)	0.845
Low/Moderate	194 (18.3)	95 (19.0)	99 (17.5)	
No Statin	601 (56.8)	280 (56.0)	321 (57.4)	
Ezetimibe use				0.980
No	707 (66.8)	334 (66.8)	373 (66.7)	
Yes	352 (33.2)	163 (33.7)	186 (33.3)	
Smoker (active)				0.279
No	872 (82.3)	405 (81.0)	467 (83.5)	
Yes	187 (17.7)	95 (19.0)	92 (16.5)	
Diabetes				0.715
No	727 (68.7)	346 (69.2)	381 (68.2)	
Yes	332 (31.4)	154 (30.8)	178 (31.8)	
Hypertension				0.354
No	320 (30.2)	158 (31.6)	162 (29.0)	
Yes	739 (69.8)	342 (68.4)	397 (71.0)	

ASCVD = atherosclerotic cardiovascular disease, LDL-C = LDL-cholesterol, CAD = coronary artery disease, CVD = cerebrovascular disease, PAD = peripheral vascular disease, PCSK9i = PCSK9 inhibitor. Values are means ± SD or %. The LDL cholesterol values represent the baseline LDL cholesterol from before the PCSK9 inhibitor was prescribed.

**Table 2:** Trends in New PCSK9 Inhibitor Prescriptions Over Time.

Year	Eligible Patients	New PCSK9i Prescriptions (%)	Physicians in Practice	New Prescriptions per Physician
2015	17,267	45 (0.26)	93	0.48
2016	17,559	216 (1.23)	94	2.30
2017	18,035	196 (1.09)	96	2.04
2018	18,771	286 (1.52)	96	2.98
2019	19,689	316 (1.60)	97	3.26

**Table 3:** Multivariable Logistic Regression Analysis for Predicting Receiving a New PCSK9 Inhibitor Prescription After Versus Before the Price Reduction.

Parameter	Odds Ratio (95% CI)	*p*-Value
Age (per year)	0.97 (0.95–0.98)	<0.001
Female Gender	0.91 (0.69–1.19)	0.485
Insurance Type		
Medicare	reference	
Medicaid	0.92 (0.54–1.56)	0.766
Commercial	1.17 (0.84–1.61)	0.352
Out of Pocket	0.57 (0.095–3.40)	0.537
Diabetes	0.97 (0.73–1.28)	0.818
Ezetimibe use	0.97 (0.74–1.28)	0.851
Smoker (active)	0.77 (0.55–1.06)	0.113
Hypertension	1.17 (0.88–1.55)	0.284
Statin use	0.80 (0.59–1.10)	0.171
LDL-C (mg/dL)		
≥70 to 99	0.61 (0.34–1.11)	0.109
100 to 129	reference	
130 to 159	0.54 (0.30–0.99)	0.045
160 to 180	0.54 (0.29–0.99)	0.048
≥190	0.49 (0.27–0.90)	0.022

CI = confidence intervals.

## Data Availability

Not applicable.
